# C-954-15. The Financial Effects of Medicaid Cuts on US Burn Care

**DOI:** 10.1093/jbcr/irag033.147

**Published:** 2026-04-06

**Authors:** Jennifer K Shah, Clifford C Sheckter

**Affiliations:** Geisel School of Medicine at Dartmouth, NY, New York; Stanford University School of Medicine, Palo Alto, CA

## Abstract

**Introduction:**

The federal government recently passed legislation that will significantly reduce Medicaid spending through work requirements. Burns disproportionately affect the Medicaid population and are one of the costliest injuries with some large burn admissions incurring multimillion dollar hospital stays. Understanding the effects of this legislation on the economics of US burn care is crucial to continue providing treatment.

**Methods:**

All admissions with a primary burn diagnosis were extracted from the National Inpatient Sample from the 6 most recently available years (2017-2022). Charge to cost ratios were applied per the Healthcare Cost and Utilization Project and adjusted for inflation to 2025 US dollars. Facilities were stratified into profit and safety net status. Payor mix (Medicaid, Medicare, private, self-pay) was evaluated for each facility. Reductions in Medicaid enrollment were modeled using US Congressional Budget Office estimates. Projections of burn care and burn center losses over a 6-year period with 95% confidence intervals (CIs) were derived from Monte Carlo simulations using bootstrap resampling distributions. Individual burn center losses were estimated by scaling national losses and CIs according to the proportion of center volume covered by Medicaid.

**Results:**

178545 weighted burn encounters were identified of which 39 575 under 18 were excluded as children will not be affected by the new law. The change in payor mix over the past 6 years has shown a 7.0% increase in Medicaid. The total cost of burn care per year is estimated at $3 190 265 497 of which Medicaid represents $906 384 428 (28.4% of total spending). Medicaid payments to burn centers increased by 18.9% over the past 6 years adjusted for inflation (Fig. 1). 33.1% of Medicaid encounters are cared for at safety-net hospitals. Assuming the current trajectory of inpatient admissions, the overall reduction in Medicaid payments on burn care is estimated at $564 030 080 (low end) to $787 753 600 (high end). From the current baseline, this presents a 3.0% reduction in burn center funding for safety-net hospitals and 2.7% reduction in funding to non-safety net hospitals. Financial losses per burn center are modeled as a function of safety-net status and Medicaid fraction (Fig. 2).

**Conclusions:**

Burn centers will experience a significant reduction in revenue from Medicaid cuts which will be harder felt by safety net hospitals. Revenue-generating strategies and cost containment may be insufficient for financial solvency in some burn centers.

**Applicability of Research to Practice:**

All burn centers, especially those who are part of safety-net hospitals or have a high percentage of Medicaid admissions will notice a significant decline in operational income.

**Funding for the study:**

N/A.

